# Medical School Attendance

**Published:** 1915-02

**Authors:** 


### MEDICAL SCHOOL ATTENDANCE.

In an article on university registration statistics, *Science*[8] furnishes a tabulation of the attendance at thirty of the universities of the country. The editor suggests that there is a theory that universities and colleges have unusually large increases in numbers of students when national economic conditions are at a low ebb, that is, during "hard times." The registration statistics presented in the article referred to seem to bear this out. The period of depression in the early portion of 1914 was prolonged

[8] Science, Dec. 25, 1914.

by the declaration of war. Perhaps as a consequence, at least as a coincidence, three universities, Columbia, California and Pittsburgh, gained over 1,000 students each in all departments; six, Ohio State, Wisconsin, New York University, Minnesota and Pennsylvania, gained more than 500, and five others, Illinois, Nebraska, Cornell, Cincinnati and Michigan, gained over 300.

It is usually said that the increase of registration in hard times is particularly to be noted in the professional schools, apparently because business reverses make men realize that the mental capital required for professional work is of greater permanent value than the more material resources demanded for commercial and financial success. There is a noteworthy increase of registration in dentistry; the University of Pennsylvania has 663 students enrolled in its dental department; Northwestern, 578; Michigan, 318; Iowa State, 302; Minnesota, 253; Pittsburgh, 227, and Harvard, 204. The remarkable increase in all the departments of the University of Pittsburgh has brought that institution into a prominent place in the statistics, for it gained altogether 1,069 students. This gain was exclusive of any addition in numbers from attendance at summer sessions, which swelled the figures for other institutions.

The statistics referred to show that our universities now begin to rival even the largest of the foreign universities in the numbers of students registered. During the present year, and while the war lasts, there will of course be much more educational activity in this country than in any of the countries of Europe. The following universities now have more than 5,000 students in attendance: Columbia, 11,294; California, 8,180; Chicago, 7,131; Wisconsin, 6,696; Pennsylvania, 6,505; Harvard, 6,411; Michigan, 6,319; New York University, 6,142; Cornell, 5,939; Illinois, 5,664.

In regard to the number of medical students, however, the increase shown by the statistics can not be taken as an index of the enrollments in all medical schools. Statistics gathered by the Council on Medical Education show that practically all of the medical colleges which had adopted higher entrance requirements prior to the beginning of the present session had increased enrollments. Instead of being due to hard times, however, this increase was doubtless influenced by the extensive agitation in recent years pointing to the need of a better preliminary education for students who were to enter on the study of medicine. A more immediate factor, perhaps, is that thirty-seven other medical colleges at the beginning of the present session put into effect for the first time the requirement of one year of collegiate work, in addition to the usual high school education. These thirty-seven colleges naturally had smaller enrollments which more than offset the increased registrations in the colleges referred to above. Incidentally, even the increased registration in dental colleges may have been due less to hard times than to the higher entrance requirements of these thirty-seven medical colleges.

Altogether, instead of there being an increase in the enrollments of medical colleges during this session, it is quite evident from the figures gathered by the Council on Medical Education that there will be a decided decrease. Of about sixty-five medical colleges from which reports have been received, enrollments show that this decrease will be about 8 per cent., as compared with from 3 to 6 per cent. in each of the last few years. The raising of the standards for admission to medical colleges, therefore, has had a favorable effect in decreasing the number of those who take up medicine as a life work. The number has been reduced to nearly the normal supply for this country, and that supply will be much better qualified. —Editorial, *Journal A. M. A.*

